# Drug-Inclusive Inorganic–Organic Hybrid Systems for the Controlled Release of the Osteoporosis Drug Zoledronate

**DOI:** 10.3390/molecules27196212

**Published:** 2022-09-21

**Authors:** Maria Vassaki, Savvina Lazarou, Petri Turhanen, Duane Choquesillo-Lazarte, Konstantinos D. Demadis

**Affiliations:** 1 Crystal Engineering, Growth and Design Laboratory, Department of Chemistry, University of Crete, 71003 Heraklion, Greece; 2 School of Pharmacy, University of Eastern Finland, Biocenter Kuopio, P.O. Box 1627, 70211 Kuopio, Finland; 3 Laboratorio de Estudios Cristalográficos, IACT, CSIC-Universidad de Granada, 18100 Granada, Spain

**Keywords:** osteoporosis, bisphosphonates, zoledronate, metal phosphonates, hybrid materials, controlled release, MOFs, strontium, barium

## Abstract

Bisphosphonates (BPs) are common pharmaceutical treatments used for calcium- and bone-related disorders, the principal one being osteoporosis. Their antiresorptive action is related to their high affinity for hydroxyapatite, the main inorganic substituent of bone. On the other hand, the phosphonate groups on their backbone make them excellent ligands for metal ions. The combination of these properties finds potential application in the utilization of such systems as controlled drug release systems (CRSs). In this work, the third generation BP drug zoledronate (ZOL) was combined with alkaline earth metal ions (e.g., Sr^2+^ and Ba^2+^) in an effort to synthesize new materials. These metal–ZOL compounds can operate as CRSs when exposed to appropriate experimental conditions, such as the low pH of the human stomach, thus releasing the active drug ZOL. CRS networks containing Sr^2+^ or Ba^2^ and ZOL were physicochemically and structurally characterized and were evaluated for their ability to release the free ZOL drug during an acid-driven hydrolysis process. Various release and kinetic parameters were determined, such as initial rates and release plateau values. Based on the drug release results of this study, there was an attempt to correlate the ZOL release efficiency with the structural features of these CRSs.

## 1. Introduction

Bisphosphonates (BPs) have been introduced to the pharmaceutical market since the late 1970s as drugs for disorders of calcium metabolism [1]. BPs are chemically stable structural analogs of inorganic pyrophosphate. They possess a carbon in the place of the bridging O of pyrophosphate. This endows them with resistance to hydrolysis and high affinity for hydroxyapatite (HAP), the principal natural mineral component of bone and teeth [2].

**Figure 1:**
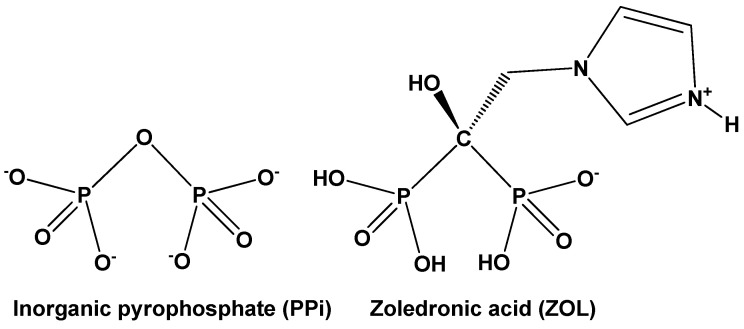
Schematic structures of inorganic pyrophosphate (PPi, left, in its fully deprotonated form) and zoledronic acid (right, in its zwitterionic form).

Zoledronate (ZOL, Figure 1) is a third generation N-containing BP drug, 5000 times more potent than etidronate, a 1st generation drug. It is administered as a solution or in tablet form (as zoledronic acid) under several commercial names (e.g., Zometa^®^). It exhibits an affinity constant *K*_app_ = 34.7 ± 1.8 × 10^5^ M^−1^ for hydroxyapatite [3]. The binding affinity determines drug absorption and retention by the bone. These features affect its osteoclast inhibition potency. ZOL was approved in 2001 (by the FDA) for the treatment of several bone-related disorders (e.g., osteoporosis, high blood calcium due to cancer, bone breakdown due to cancer, Paget’s disease of bone and Duchenne muscular dystrophy).

In general, BPs are administered orally and in some cases via injection. Their bioavailability is very low (depending on the individual BP) and only a small portion of the drug is absorbed by the intestine, distributed via blood in the body and finally reaching bone. Hence, the therapeutic dosage is usually increased in order to achieve effective patient treatment, resulting in several side effects, such as hypocalcemia, osteomyelitis, osteonecrosis of the lower jaw, “flu”-like symptoms, bone pain, and gastro-intestinal, ocular and renal side effects [4].

One strategy to reduce side effects is the fabrication of controlled release systems (CRSs) that could demonstrate release of the active BP drug in a predictable and controlled way. One approach is to view BPs as organic ligands for metal ions. The presence of the anionic phosphonate groups on the BP backbone endows them with strong affinity for metal ions in aqueous solutions [5]. Hence, metal-containing hybrid materials were constructed by combining BPs with biologically acceptable metal ions such as Mg^2+^ and Ca^2+^, or their surrogates Sr^2+^ and Ba^2+^. Some biological applications of coordination compounds and metal organic frameworks (MOFs) included their evaluation as CRSs under different conditions that mimic the conditions in human body (e.g., the gastrointestinal tract) [6].

Recently, we initiated a systematic study of metal-containing coordination polymers in which the linker is an actual BP drug. The scope of this study includes the synthesis of such metal–BP systems, their full structural elucidation, and the evaluation of their ability to act as sources of the BP drug when exposed to appropriate conditions. For example, Mg^2+^– and Ca^2+^–containing complexes and coordination polymers of various structural motifs were synthesized with four BP drugs (etidronate, pamidronate, alendronate and neridronate) and were studied as CRSs [7]. Their metal–O(phosphonate) coordination bonds can undergo hydrolysis (at low pH), leading to the controlled release of the active BP drug. These were coined “self-sacrificial MOFs” because their decomposition must precede drug release. The coordination of the BP drug by the metal ions resulted in substantially reduced initial release rates and lower final % release compared to the respective control system (with “free” drug and no metals). Recently, the 3^rd^ generation anti-osteoporotic drug risedronate (RIS) was used for the synthesis of two new coordination polymers, namely [Ca(RIS)(H_2_O)]_n_ (Ca–RIS) and [Sr(RIS)(H_2_O)]_n_ (Sr–RIS) [8]. These two novel compounds were physicochemically and structurally characterized and were also evaluated for their RIS release features under acidic conditions (pH = 1.3) that mimic the human stomach. It was found that the drug release profiles Ca- and Sr–RIS (in the presence of linear polyethyleneimine polymer as drug solubility enhancer) were 4–5 times faster than the “free” RIS system.

In this paper, the synthesis and characterization of two CRSs for the drug ZOL are presented, namely Sr–ZOL and Ba–ZOL. In addition, the controlled release of ZOL is studied in these systems and compared to the “free” ZOL system (no metals). To the best of our knowledge, this is the first systematic controlled release study of the ZOL drug.

## 2. Results

### 2.1. Synthesis and Characterization of (Sr/Ba)–ZOL Compounds

**Figure 2:**
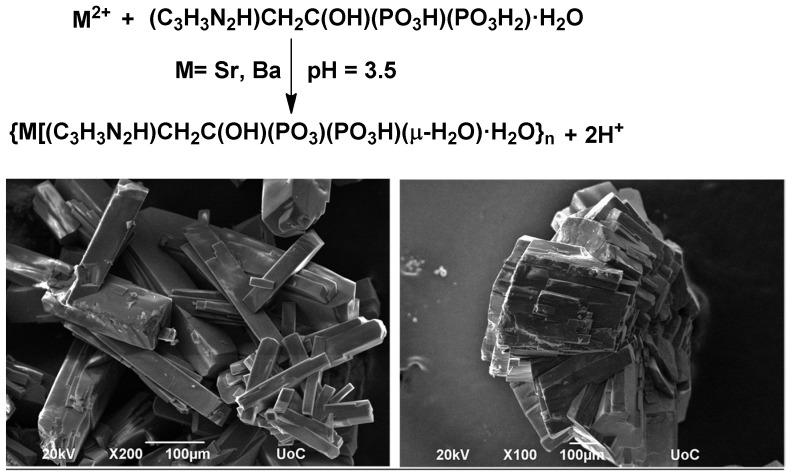
Reaction scheme for the synthesis of (Sr/Ba)–ZOL (**upper**) and SEM images of single crystals of Sr–ZOL (**lower left**) and Ba–ZOL (**lower right**).

The new (Sr/Ba)–ZOL compounds were synthesized under ambient conditions in mildly acidic pH (3.5), by reacting ZOL acid monohydrate with strontium or barium chloride, respectively, Figure 2 (upper). The solution pH is crucial for isolating tractable products. Excessively low pH will cause crystallization of unreacted ZOL, whereas high pH will result in fast product precipitation that is usually amorphous, or with low crystallinity. Inevitably, extensive experimentation with various solution pH values must be carried out. It was found that the pH of 3.5 is optimal for the isolation of pure, monophasic products. These syntheses yielded single crystals suitable for X-ray diffraction studies. The crystals were isolated and studied by scanning electron microscopy as well. The morphology and the size of the crystals of the two compounds are shown in Figure 2.

The (Sr/Ba)–ZOL compounds were studied by ATR-IR spectroscopy (see Figure S1, Supplementary Materials). The vibrational frequencies between 2600–3200 cm^−1^ are attributed to N-H stretching vibration and C-H symmetric and antisymmetric stretching vibrations of the heteroaromatic ring of imidazole [9]. Τhe spectral region 900–1200 cm^−1^ is complex and includes several characteristic vibrations related to the –PO_3_ moieties of ZOL [10]. The other frequencies between 1440–1650 cm^−1^ are assigned to the C=C and C=N stretching vibrations of the heterocyclic aromatic ring [9]. The range 700–850 cm^−1^ is associated with out of plane bending of C–H of imidazole [10].

### 2.2. Powder and Single Crystal X-ray Diffraction Studies of (Sr/Ba)–ZOL Compounds

Bulk solid products of the (Sr/Ba)–ZOL compounds were studied by powder X-ray diffraction to ensure that they were pure and monophasic. Comparison of the calculated (from the crystal structure determination, see below) and measured X-ray diffraction diagrams ensured that the samples were single phases (Figures S2–S4, Supplementary Materials).

Suitable crystals for single crystal X-ray diffraction and structure determination were obtained by syntheses at ambient conditions (see Materials and Methods). The crystallographic data of the compounds Sr–ZOL and Ba–ZOL are summarized in Table 1 and their cif files are provided as Supplementary Materials.

**Figure 3:**
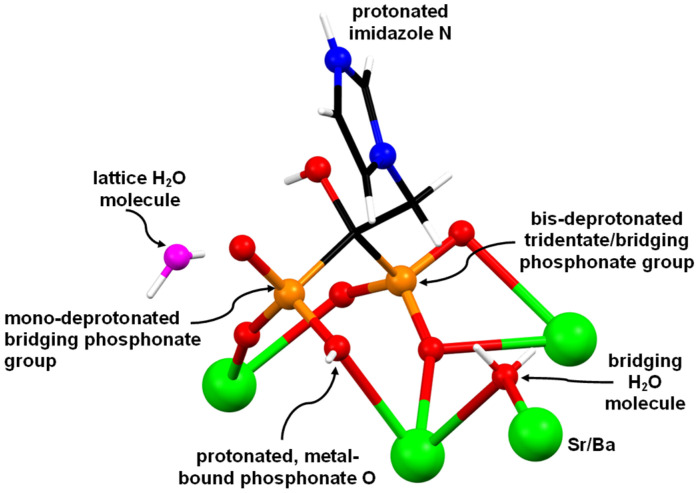
Depiction of the environment of the ZOL ligand in the structure of compounds Sr–ZOL and Ba–ZOL. Color codes: metal centers, green; P, orange; O, red; C, black; N, blue; H, white; lattice H_2_O, magenta.

The starting material, ZOL acid, exists as a zwitterion, because the “external” N atom is protonated, while one phosphonate group is singly deprotonated [11]. However, in the structures of Sr–ZOL and Ba–ZOL it exhibits a total charge of “−2” to counterbalance the “+2” charge of the metal cation. This means that one of the phosphonate groups (P1) is mono-deprotonated, whereas the other (P2) is bis-deprotonated. Hence, ZOL behaves as a zwitterion in the Metal-ZOL structures. Sr–ZOL and Ba–ZOL are principally isostructural, with only minor differences in the hydrogen-bonding scheme. Hence, structural details of the Ba-ZOL compound will be discussed. A representation of the basic structure of Sr/Ba-ZOL is shown in Figure 3. Each ZOL ligand coordinates to three metals, and hence, Sr/Ba-ZOL are 2D coordination polymers. The mono-deprotonated phosphonate (P1) bridges two neighboring metal centers by using two O atoms (O1 and O2), while the third O atom (O3) remains non-coordinated. Interestingly, O1 is the protonated oxygen with the longest P-O bond length 1.534 Å. The fully deprotonated phosphonate (P2) also bridges two metal centers, but in a very different fashion. Two of the O atoms (O4 and O5) chelate a metal center (forming a 4-membered ring), while the third O atom (O6) binds a neighboring metal. The –OH group and the imidazole ring (both connected to the central C) are non-coordinating.

The P-O bond lengths are influenced by the protonation state of ZOL, but also by the coordination to a metal center. For example, in the structure of free ZOL (anhydrous) there are two “long” P-O bonds (1.548 Å and 1.554 Å) that correspond to the fully protonated –P-O-H moieties and a “short” P-O bond for the P=O moiety (1.498 Å) [12]. In the same structure, there is a mono-deprotonated phosphonate group (-PO_3_H^−^) that has been generated by internal protonation of one of the N atoms in the imidazole ring. There are also two kinds of P-O bonds for the -PO_3_H^−^ group, a “short” P=O bond (1.505 Å) and two “long” P-O bonds (1.555 Å and 1.527 Å). The former is the P-O(H) bond, and the latter is the P-O^−^ bond. Hence, it appears that the P-O bond is somewhat shortened upon deprotonation. The case of the Sr/Ba–ZOL structures is more complicated because there is metal coordination involved. We will examine the Sr–ZOL case as the example and discuss the two crystallographically and chemically different phosphonate groups (P1 and P2). The phosphonate group P2 is fully deprotonated and coordinated asymmetrically to three Sr centers (with monodentate, chelating and bridging modes). P2 displays two “short” P-O bonds (P2-O4 1.505 Å and P2-O5 1.516 Å) and one “long” bond P2-O6 1.555 Å. Hence, the “short” P-O bonds are reminiscent of the P=O and P-O^−^ moieties. We assign the presence of the “long” bond to the particular coordination mode of the ZOL ligand. The phosphonate group P1 is mono-deprotonated and coordinated asymmetrically to two Sr centers (with monodentate modes, but the entire phosphonate bridges two Sr centers). P1 displays two “short” P-O bonds (P1-O2 1.505 Å and P1-O3 1.510 Å) and one “long” bond P1-O1 1.559 Å. The two “short” P-O bonds, of almost equal length, must be the result of delocalization of the negative charge (−1) over the P=O and P-O^−^ moieties. The O atom of the “long” P-O bond is protonated (and coordinated to the Sr), consistent with the length of the bond.

**Figure 4:**
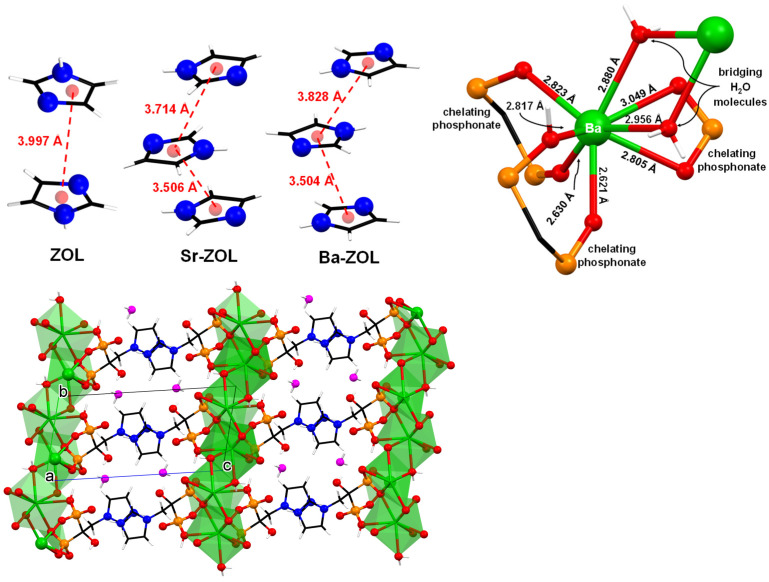
Various views of the crystal structures of Sr/Ba–ZOL: comparative π−π stacking interactions (**upper left**). The coordination sphere of the of Ba^2+^ center with Ba–O bond distances (**upper right**). Packing of three 2D layers along the b-axis (**lower**). Color codes: Βa, green; P, orange; O, red; C, black; N, blue; H, white, lattice H_2_O, magenta.

In both compounds Sr/Ba–ZOL, the coordination sphere of the M^2+^ center can be described as a biaugmented trigonal prism (*C*_2v_, based on SHAPE analysis), while they form six coordination bonds with oxygen atoms of three different ZOL ligands and two bonds with water molecules; see Figure 4. A list of all types of interactions in the structures of ZOL acid monohydrate, Sr-ZOL and Ba-ZOL, can be found in Table 2. Both these metal-bound H_2_O molecules are positioned *cis* to each other and bridge neighboring metals. The Ba^2+^ cation chains are arranged in such a way that they lie parallel to the b-axis (Figure 4). The Ba–O bond distances (Figure 4) are between 2.621 Å and 3.049 Å. There are two types of interactions between the chains, hydrogen bonds and π−π stacking interactions. The latter are shown in Figure 4 together with those in the structure of ZOL acid monohydrate. They occur between the imidazole rings that are positioned in the interlayer space. 

There is one lattice water molecule (O9) per asymmetric unit in the structure of the M-ZOL compounds. It is situated in the interlayer space, but close to the metal-phosphonate layer. It forms three hydrogen bonds, two with phosphonate O atoms from neighboring units (O9···O5 2.761 Å and O9···O3 2.698 Å) and one with the C–OH group (O9···O6 2.644 Å). The protonated N of the imidazole ring forms a hydrogen bond with an uncoordinated O of the phosphonate P1 (O3···N2 2.625 Å).

### 2.3. Controlled Release Study of “Free” ZOL, Sr–ZOL and Ba–ZOL

**Figure 5:**
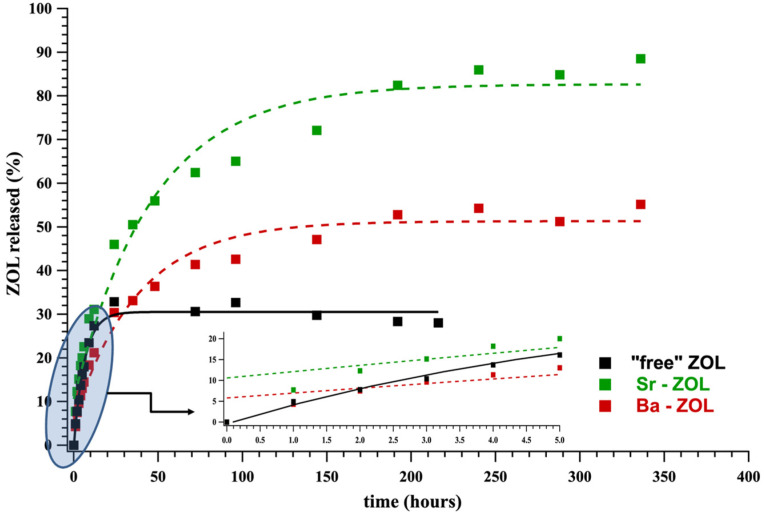
ZOL release curves from Sr– and Ba–ZOL containing tablets and comparison with the “free” ZOL system.

Three controlled release systems (CRSs) were evaluated in the form of tablets: (a) the “free” drug ZOL acid monohydrate, (b) Sr–ZOL, and (c) Ba–ZOL. In each case, the active agent (the “free” ZOL drug and its metal–ZOL coordination compounds) was mixed and ground with the appropriate excipients in the solid form, and these powders were pressed into tablets. These tablets were immersed into acidic solutions and aliquots were withdrawn at specific time intervals. The detailed protocols for tablet preparation, sampling and drug quantification are described in detail in Section 4.4 and Section 4.5. Under the acidic conditions of the drug release experiments, hydrolysis of the metal–O bonds occurs that leads to the degradation of the crystal lattice and the release of ZOL into the acidic medium. The drug release was quantified by ^1^H NMR spectroscopy and the results were plotted in graphs as “% ZOL released” vs. time (in hours). The release curves for all systems are shown in Figure 5 and some kinetic parameters are collected in Table 3.

The Ba–ZOL system exhibits an initial rate of 0.33 µmol/min, slightly lower than the “free” ZOL system, 0.39 µmol/min. Furthermore, 134.1 hours are needed for the Ba–ZOL system to reach an equilibrium (plateau) of 50%, while the t_p_ is only 25.5 h for the “free” ZOL to reach its plateau value of 30%. We assigned the slower release kinetics of ZOL from the Ba–ZOL system to the hydrolysis of the Ba-O bonds, a requirement for the detachment of ZOL molecules from the coordination network and their subsequent dissolution into the aqueous phase. The Sr–ZOL system displays an enigmatic behavior. First, its initial rate (0.50 µmol/min) is higher than the “free” ZOL system (0.39 µmol/min) and the Ba–ZOL system (0.33 µmol/min). Secondly, its plateau value of 80% is higher than that of the Ba–ZOL system, and it needs an even longer time (153.2 h) to reach it. Interpretation of these results is presented in the Discussion section.

**Figure 6:**
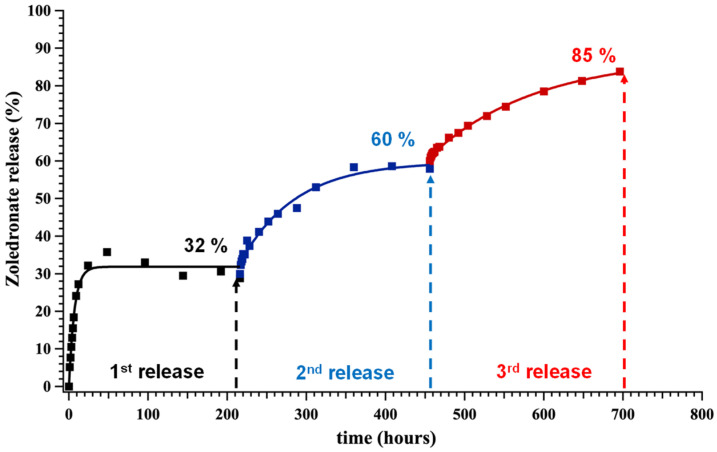
Cumulative, step-wise release of ZOL from the “free” ZOL system.

Furthermore, we wanted to investigate whether a tablet, after reaching the plateau value is still active in release of the active drug. For this purpose, a series of consecutive release steps were set up in the following way. The “free” ZOL tablet was selected for this study. After the first plateau of 32% was established (25.5 h), the release experiment was allowed to proceed for up to ~210 h to ensure equilibrium. At that point, the aqueous phase was replaced with a “fresh” one (pre-acidified at pH 1.3), thus signifying the onset of a second release (second step). The second release was allowed to proceed up to the 450th hour, reaching a cumulative plateau value of 60%. By following the same methodology, a third step was initiated immediately after, reaching a cumulative plateau value of 85%. The results are shown in Figure 6. A fourth step was also done (results shown in Figure S5, Supplementary Materials), allowing the system to release the ZOL quantitatively. These results prove that the ZOL–containing tablet, if placed under the appropriate conditions, can release 100% of the active ingredient and the entire quantity of ZOL is solvent-accessible. Such a step-wise behavior has been noted in the controlled release of etidronate and pamidronate from silica-based hydrogels [13]. 

## 3. Discussion

One of the main advantages of using crystalline metal–bisphosphonate materials as controlled release systems is the knowledge of the precise identity of the drug-releasing compound. There are two basic requirements for this: (a) the synthetic protocols must yield monophasic products, and (b) the crystal structure of the product must be known. A successful synthesis outcome involves extensive experimentation with variables such as solution pH, reactant concentrations and molar ratios and temperature (and pressure, in the case of hydrothermal synthesis). For these Sr/Ba–ZOL systems, the optimum synthesis pH was 3.5, which yielded crystalline solids with Sr/Ba–ZOL molar ratio 1:1.

The published crystal structures containing the BP zoledronate include its “free” forms, its organic salt forms and its metal-containing forms. The “free” forms include two polymorphs (moniclinic and triclinic) of anhydrous zoledronic acid [12], zoledronic acid monohydrate [14], and zoledronic acid trihydrate [15,16]. The organic salt forms of zoledronate include the cytosinium zoledronate trihydrate [17], the dicyclohexylammonium zoledronate [18] and the bis(ammonium) zoledronate [19]. The metal-containing forms of zoledronate include structures with metal centers, such as K^+^ [20], Zn^2+^ [21], Mn^2+^ [22], Fe^3+^ [22], Co^2+^ [23], Ni^2+^ [23], and Cu^2+^ [24].

ZOL release experiments were carried out at pH 1.3. This value was selected to mimic the pH of the human stomach. We are aware of the fact that the stomach fluid is a much more complicated system, which cannot be fully simulated in the laboratory. However, based on the fact that metal phosphonate compounds are unstable at such low pH, it was decided that pH is the principal factor for the acid-driven hydrolysis of the M-O(phosphonate) bonds and this approach is sufficient for a “proof-of-concept”. ^1^H NMR spectroscopy was used for the quantification of ZOL in the supernatant phase. It was preferred over ^31^P NMR, which is time consuming and impractical for this particular application. We have successfully used ^31^P NMR for quantification of drugs without any hydrogens, for example clodronate (unpublished results). The controlled release curves of all systems studied here are shown in Figure 5 and Figure 6. The results obtained here show that the metal-containing ZOL systems exhibit similar (Ba–ZOL) or higher (Sr–ZOL) initial release rates than the “free” ZOL system (no metals). This observation is in contrast to what was noted in our previous work [7] for other BP drugs, in which the “free” drug was released much faster than the metal-containing drugs. The results obtained herein appear to be counterintuitive because the hydrolysis of the M-O bonds in the Sr–ZOL and Ba–ZOL systems does not appear to slow down the release kinetics of the active ZOL.

The release features of the “free” ZOL system may be related to the stability of the crystal lattice of the compound. The water solvent must overcome several stabilizing interactions. There are medium-strength π–π interactions between the imidazole rings (centroid-to-centroid distance 3.997 Å). The presence of one lattice water further stabilizes the crystal structure because hydrogen bonds are formed with the phosphonate groups. Each ZOL molecule forms 11 hydrogen bonds in total (phosphonate groups and imidazole ring), Table 2. As mentioned above, ZOL exists as a zwitterion in its crystal lattice, so, effectively it may behave as a “dipole”. Hence, its crystal packing is not only a result of hydrogen bonding interactions, but also of ionic interactions, the latter acting to further stabilize the crystal lattice.

Both metal–ZOL compounds can be described as 2D coordination polymers, propagated by various phosphonate bridging modes. There are π–π interactions (Figure 4) between the ZOL imidazole rings and the centroid-to-centroid distances range from 3.504 Å to 3.828 Å. There are 11 H-bonding interactions (per molecular unit) in Sr–ZOL and 9 in Ba–ZOL. The coordination geometry of the metal centers is the same in the two compounds. The Sr–ZOL displays the fastest initial rate. Careful examination of its crystal structure reveals that the layers are not stabilized by H-bonding, and for that reason, they can be readily exfoliated by the water solvent. Thus, the Sr–O bonds are exposed to the acidic medium and could be easily hydrolyzed.

Both Sr–ZOL and Ba–ZOL compounds are isostructural, with only minor differences in the hydrogen-bonding scheme. According to the argument that Sr-O(phosphonate) bonds are stronger (and harder to hydrolyze) than the corresponding Ba-O(phosphonate) bonds, the Sr–ZOL system should demonstrate slower release kinetics than the Ba–ZOL system. However, this is not the case, as evidenced by the parameters in Table 3. The only viable explanation for this phenomenon lies with the morphology and size of the metal–ZOL particles. The Sr–ZOL particles are needle-like, in contrast to crystal aggregates in Ba–ZOL, therefore exposing a much larger surface to the solvent. Since the dissolution process is surface-dependent, it is reasonable to expect that Sr–ZOL should undergo dissolution much more effectively (and rapidly) than Ba–ZOL. Furthermore, the particle size of Sr–ZOL is much smaller than that of Ba–ZOL. 

## 4. Materials and Methods

### 4.1. Materials

All reagents that were utilized as sources of metal ions were from commercial sources. Strontium chloride hexahydrate (SrCl_2_·6H_2_O) and barium chloride dihydrate, (BaCl_2_·2H_2_O) were purchased from Sigma-Aldrich (St. Louis, MO, USA). The tablet excipients lactose (Serva), cellulose (Merck) and silica (Alfa-Aesar) were from commercial sources. Deionized (DI) water was used in all experiments and was produced from a laboratory ion exchange column. 

### 4.2. Instrumentation

**Scanning electron microscopy.** Elemental analyses and SEM images of the morphology of the metal–BPs collected with a JOEL JSM-6390LV electron microscope.

**Single crystal X-ray diffraction.** Measured crystals were prepared under inert conditions immersed in perfluoropolyether as protecting oil for manipulation. Suitable crystals were mounted on MiTeGen Micromounts™, and these samples were used for data collection. Data were collected with a Bruker D8 Venture diffractometer with graphite monochromated CuKα radiation (λ = 1.54178 Å). The data were processed with APEX3 suite [25]. The structures were solved by intrinsic phasing using the ShelXT program [26], which revealed the position of all non-hydrogen atoms. These atoms were refined on F^2^ by a full-matrix least-squares procedure, using the anisotropic displacement parameter [27]. All hydrogen atoms were located in different Fourier maps and were included as fixed contributions riding on attached atoms with isotropic thermal displacement parameters 1.2- or 1.5-times those of the respective atom. The Olex2 software was used as a graphical interface [28]. Molecular graphics were generated using mercury [29]. The crystallographic data for the reported structures were deposited with the Cambridge Crystallographic Data Center as supplementary publication nos. CCDC 2195677 and 2195679. Copies of the data can be obtained free of charge at http://www.ccdc.cam.ac.uk/products/csd/request.

**Powder X-ray diffraction.** The powder X-ray diffraction (XRD) patterns were performed on PANalytical X’Pert Pro diffractometer, a configuration of the Bragg–Brentano, equipped with monochromator Ge(111) (Cu Κ_α1_) and detector X’Celerator.

### 4.3. Synthetic Protocols

**Synthesis of (1-hydroxy-2-(1H-imidazol-1-yl)ethane-1,1-diyl)bis(phosphonic acid), zoledronic acid monohydrate(ZOL).** ZOL was synthesized by following a previously reported method with some modifications [30]. A mixture of 1-imidazoleacetic acid (10 g, 0.079 mol), phosphorous acid (6.5 g, 0.079 mol), and methanesulfonic acid (25 mL) was heated to 65–70 °C followed by addition of PCl_3_ (13.8 mL, 21.7 g, 2.0 eq) for over 0.5 h under nitrogen atmosphere. After the PCl_3_ addition, the mixture was stirred at 65–70 °C overnight and cold distilled water (70 mL) was added to a cooled solution with vigorous stirring. After refluxing overnight, the reaction mixture was allowed to cool to room temperature and EtOH (140 mL) was added by stirring and the mixture was left at room temperature for a couple days. The formed solids were collected by filtration, washed with EtOH and finally with acetone and dried under reduced pressure. The final product was obtained as a white solid (11.9 g, 52% yield). ^1^H NMR (D_2_O): δ 7.70 (s, 1H), 7.21 (s, 1H), 6.84 (s, 1H), 4.44 (t, 2H, ^3^*J_HP_* = 9.7). ^13^C NMR (D_2_O, CD_3_OD as ref.) δ 141.1, 126.8, 123.9, 76.2 (t, ^1^*J*_CP_ = 131.5, P-*C*-P), 53.2 (t, ^2^*J_CP_* = 3.4). ^31^P NMR (D_2_O) δ 16.4. Peak assignments can be found in the Supplementary Materials. NMR data were consistent to those reported in the literature [30].

**Synthesis of M–ZOL, {{M[(C_3_H_4_N_2_)CH_2_C(OH)(PO_3_H)(PO_3_)(H_2_O)]}·H_2_O}_n_(M = Sr, Ba).** ZOL acid monohydrate (27 mg, 0.1 mmol) and either SrCl_2_·6H_2_O (13 mg, 0.05 mmol) or BaCl_2_·2H_2_O (12 mg, 0.05 mmol) were dissolved in ~ 10 mL DI H_2_O under stirring until fully dissolved. The solution pH was adjusted to 3.5 (using stock solutions of NaOH and HCl, as needed). The final mixture was left under quiescent conditions for solvent evaporation. After 7–10 days (depending on ambient temperature) a colorless crystalline product formed, it was isolated by filtration, rinsed with DI water and left to dry under air. Bulk product purity was confirmed by powder X-ray diffraction (comparison of the calculated and experimental powder patterns) and elemental analysis. Yield: For Sr-ZOL 17 mg (43%), for Ba-ZOL 20 mg (45%). Elemental analysis (%): For Sr-ZOL, calcd. for {{Sr[(C_3_H_4_N_2_)CH_2_C(OH)(PO_3_H)(PO_3_)(H_2_O)]}·H_2_O}_n_, M.W. 393.73: C 15.24; H 3.05; Ν 7.11. Found: C 14.94; H 3.26; N 7.09. For Ba-ZOL, calcd. for {{Ba[(C_3_H_4_N_2_)CH_2_C(OH)(PO_3_H)(PO_3_)(H_2_O)]}·H_2_O}_n_, M.W. 443.43: C 13.53; H 2.71; Ν 6.31. Found: C 13.31; H 2.83; N 6.37. 

### 4.4. Preparation of Tablets for ZOL Release

Tablets were prepared by mechanical mixing of ground powders (with a mortar-and-pestle) of the drug component (850 μmol of ZOL content) and three commonly used excipients, i.e., lactose, cellulose and silica. Subsequently, a tablet was prepared by applying 10 tons of pressure in a hydraulic press. The tablet total weight was 1.000 g. Identical tablets that contained equimolar amounts of the metal–BPs (Sr–ZOL and Ba–ZOL) and the three excipients were fabricated. The quantities used in the tablets are shown in Table 4.

### 4.5. Quantification of ZOL

Each tablet described above was immersed in a glass beaker containing 50 mL of deionized water whose pH was adjusted to 1.3, using hydrochloric acid. The ZOL-containing tablet (as prepared above) was placed in a plastic net and was immersed into the solution (50 mL of deionized water whose pH was adjusted to 1.3 using hydrochloric acid) just above the stirring bar. Mild stirring was applied to ensure solution homogeneity. Aliquots of the solution were withdrawn (sample volume 350 μL) every hour for the first 6 hours, then every 3 h until the 12th hour, and then every 12 h until the 48th h of the release experiment. After the 48th h, samples were withdrawn every 24 h or every 48 h or longer, if necessary. Each sample was placed in an NMR tube, and then the D_2_O standard solution (150 μL) was added. The concentration of the D_2_O TSP standard solution was 4.337 μmol. Quantification of ZOL concentration in each sample was achieved by peak integration (singlet at 8.75 ppm, which is the H on the C between the two N atoms of the imidazole ring) in the ^1^H NMR spectrum and its comparison to the peak of the TSP standard solution peak [-Si(CH_3_)_3_]. For representative ^1^H NMR spectra, see Figures S6 and S7, Supplementary Materials. Initial rates were calculated based on the initial linear portion of the curve (see Figure S8, Supplementary Materials).

## 5. Conclusions

The main findings of the present study are as follows:

(1) Two novel coordination polymers containing the alkaline-earth metal ions Sr^2+^ and Ba^2+^ and the anti-osteoporotic drug ZOL were synthesized and structurally characterized.
(2) Sr–ZOL and Ba–ZOL are isostructural 2D coordination polymers.
(3) Both Sr–ZOL and Ba-ZOL were utilized as controlled release systems (excipient-containing tablets) of the active drug ZOL in conditions that mimic the human stomach (pH = 1.3).
(4) The drug release profiles of Sr–ZOL and Ba–ZOL were compared to that of “free” ZOL (absence of metals). Contrary to the working hypothesis, it was found that the release of ZOL is not delayed compared to the “free” ZOL system. In fact, in the case of Sr–ZOL, it is accelerated.
(5) This behavior was rationalized based on the structural idiosyncrasies of each system. The overall drug release profile for each system was the result of several structural factors, such as presence or absence of π–π interactions between the ZOL imidazole rings, H-bonding interactions and strength of the metal–O(phosphonate) bonds. However, it seems that the governing factor for Sr–ZOL releasing the active drug more rapidly than Ba–ZOL is the particle size and morphology.

Based on these results, the role of the metal cation in such coordination polymers apparently influences both the initial drug release rates and the final plateau release value. Hence, with proper selection of the metal ion, these features can be controlled (increased or decreased, at will). Further research efforts along these lines are underway in our group that build upon results obtained and knowledge built [7,8,13,31,32,33,34].

## Figures and Tables

**Table 1:** Crystal data for compounds Sr–ZOL and Ba–ZOL.

	Sr–ZOL	Ba–ZOL
**Empirical Formula**	C_5_H_12_SrN_2_O_9_P_2_	C_5_H_12_BaN_2_O_9_P_2_
***M*_r_**	393.73	443.43
**Crystal System**	triclinic	triclinic
**Space Group**	$P\;\bar{1}$	$P\;\bar{1}$
**a (Å)**	6.3648(2)	6.479(2)
**b (Å)**	6.6900(2)	6.837(2)
**c(Å)**	13.9296(5)	14.173(5)
**α (°)**	102.5690(10)	77.532(12)
**β (°)**	91.4870(10)	88.748(11)
**γ (°)**	90.1080(10)	89.021(10)
**V (Å^3^)**	578.703	612.819
**Z**	2	2
**R factor (%)**	2.98	3.80
**CCDC code**	2195677	2195679

**Table 2:** All types of interactions present in the structure of ZOL acid monohydrate, Sr–ZOL and Ba–ZOL.

Compound.	P_A_	P_B_	N	OH	Total H-Bonds ^1^	π-π Interactions	M-O Bonds (PO_3_/H_2_O/OH) ^2^	Total Interactions	Lattice H_2_O	M^2+^ Cations
**ZOL** *Molecule 1*	5	4	1	1	11	1	0	12	1	0
**ZOL** *Molecule 2*	3	6	1	1						
**Sr-ZOL**	5	4	1	1	11	2	6/2/0	21	1	1
**Ba-ZOL**	4	3	1	1	9	2	6/2/0	19	1	1

^1^ Only the intermolecular H-bonds are considered. ^2^ The OH is the substituent on the central C of ZOL.

**Table 3:** Kinetic data for the drug release from tablets with “free” ZOL, Sr–ZOL and Ba–ZOL.

	Initial Rate (μmol/min) ^1^	Plateau BP (%)	t_p_ (h) ^2^	t_½_ (h) ^3^
**“free” ZOL**	0.39	30	25.5	4.3
**Sr–ZOL**	0.50	80	153.2	24.4
**Ba–ZOL**	0.33	50	134.1	20.8

^1^ Calculated based on the initial linear portion of the curve (see inset, Figure 5). ^2^ t_p_ is defined as the time required for the plateau value to be reached. ^3^ t_½_ is defined as the time required for half of the plateau value to be reached.

**Table 4:** Quantities of active agents (free ZOL or metal–ZOL) and excipients utilized for tablet preparation.

Tablet	ZOL acid·H_2_O	Sr–ZOL *	Ba–ZOL *
MW (g/mol)	290.11	393.73	443.43
Drug system (g)	0.247	0.335	0.377
Lactose (g)	0.251	0.222	0.208
Cellulose (g)	0.251	0.222	0.208
Silica (g)	0.251	0.222	0.208

* Multiple syntheses of Sr- and Ba-ZOL were necessary to collect the required quantity (see Section 4.3).

## Data Availability

Data presented in this paper are available from the authors.

## Supplementary Materials

The following supporting information can be downloaded at: https://www.mdpi.com/article/10.3390/molecules27196212/s1: Figure S1. ATR-IR spectra of zoledronic acid, Ba-ZOL, and Sr-ZOL. Figure S2. Comparison of the calculated and measured X-ray diffraction diagrams of zoledronic acid monohydrate. Figure S3. Comparison of the calculated and measured X-ray diffraction diagrams of Ba-ZOL. Figure S4. Comparison of the calculated and measured X-ray diffraction diagrams of Sr-ZOL. Figure S5. Cumulative, step-wise release of ZOL from the “free” ZOL system. Figure S6. ^1^H, ^13^C and ^31^P NMR spectra of zoledronic acid monohydrate. Figure S7. ^1^H NMR spectra of released zoledronic acid from the Ba-ZOL and Sr-ZOL systems. Figure S8. Initial rates of drug release from “free” ZOL, Sr-ZOL and Ba-ZOL.

## Sample Availability

Samples of the compounds described herein are available from the authors.
