# Supplementary material for: Drug-Inclusive Inorganic–Organic Hybrid Systems for the Controlled Release of the Osteoporosis Drug Zoledronate
Source: Molecules. 2022 Sep 21;27(19):6212. doi: 10.3390/molecules27196212 (PMC9572319; doi:10.3390/molecules27196212)
Supplement: Supplementary file 1 [file molecules-27-06212-s001.zip › molecules-1888410-supplementary.pdf]

Supplementary Material

For

Drug-inclusive inorganic-organic hybrid systems for the controlled release of the osteoporosis drug zoledronate

By

Maria Vassaki, Savvina Lazarou, Petri Turhanen, Duane Choquesillo-Lazarte and Konstantinos D. Demadis

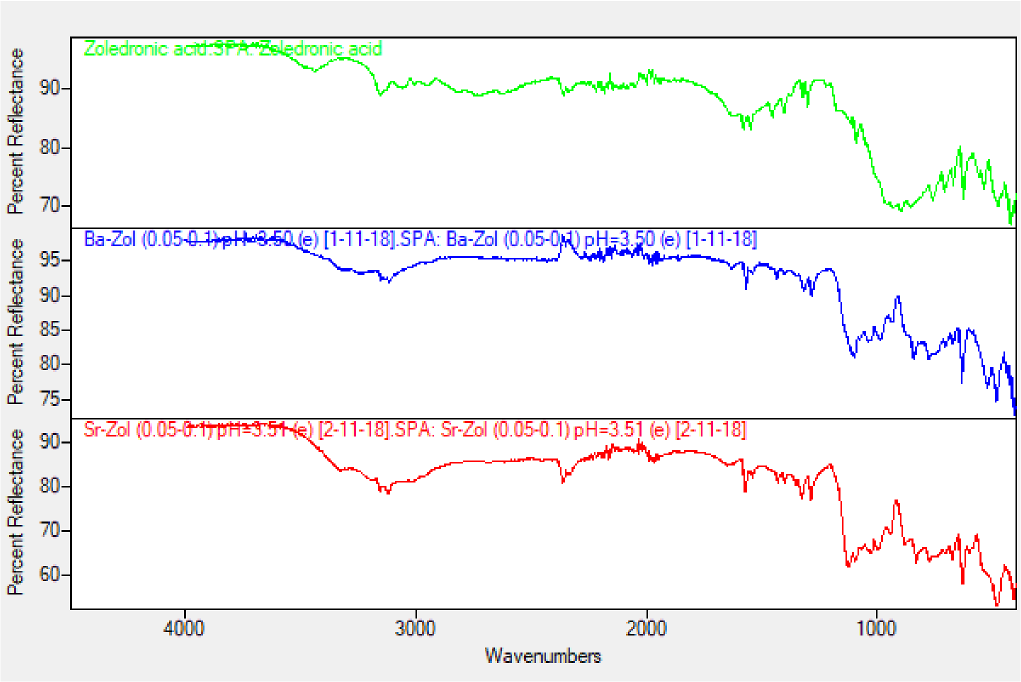

Figure S1. ATR-IR spectra of zoledronic acid (green), Ba-ZOL (blue), and Sr-ZOL (red).

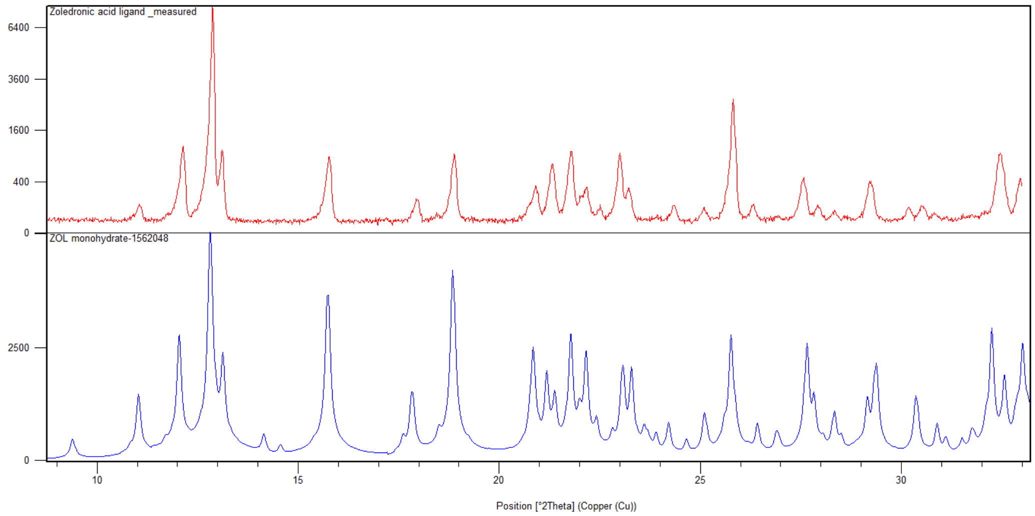

Figure S2. Comparison of the calculated (lower, blue) and measured (upper, red) X-ray diffraction diagrams of zoledronic acid monohydrate.

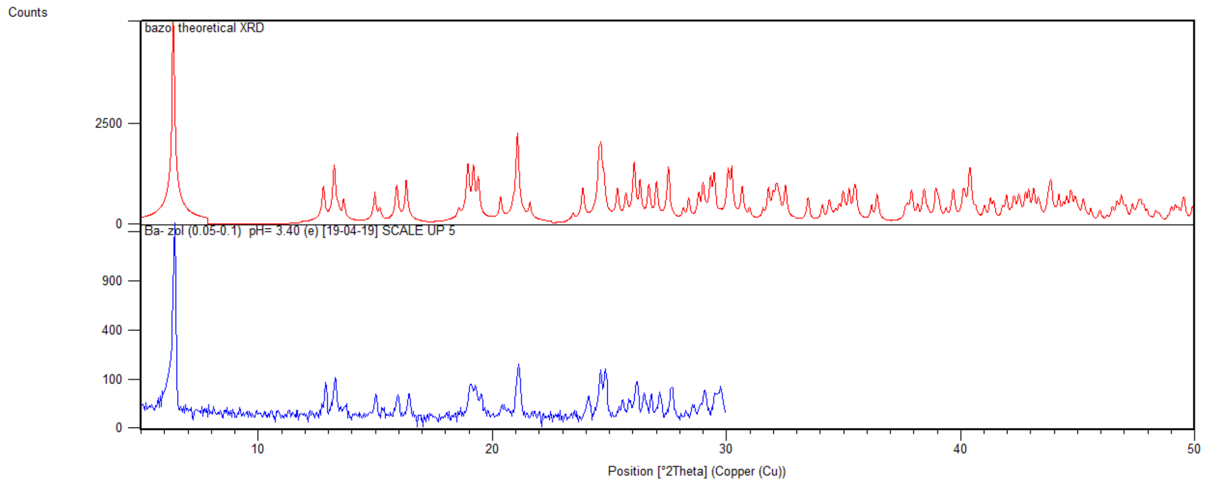

Figure S3. Comparison of the calculated (upper, red) and measured (lower, blue) X-ray diffraction diagrams of Ba-ZOL.

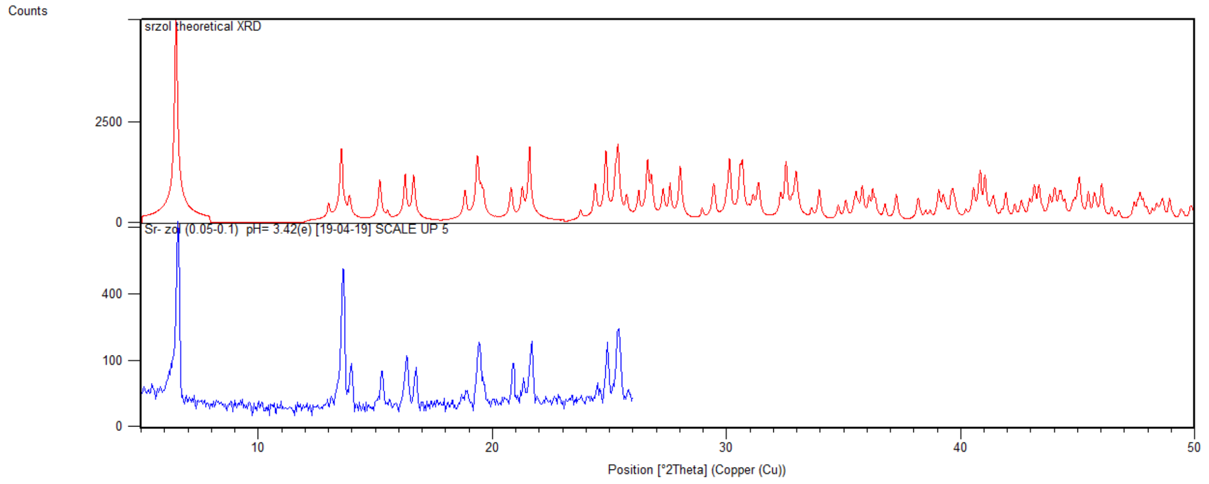

Figure S4. Comparison of the calculated (upper, red) and measured (lower, blue) X-ray diffraction diagrams of Sr-ZOL.

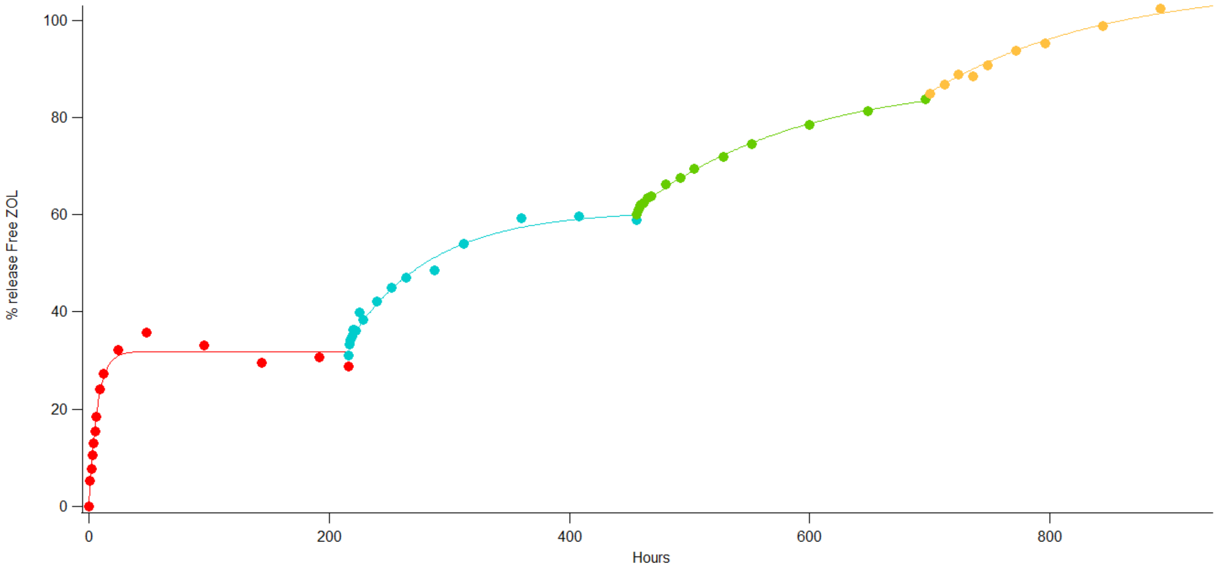

Figure S5. Cumulative, step-wise release of ZOL from the "free" ZOL system.

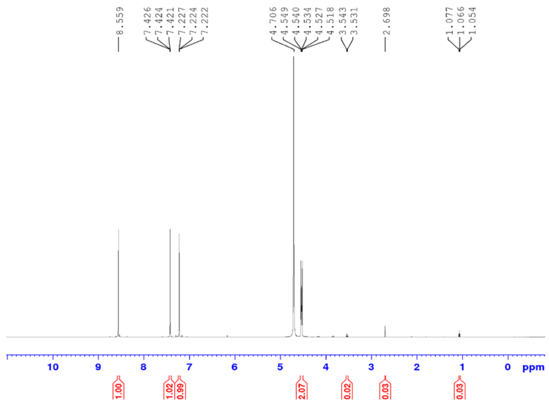
8.559
7.426
7.424
7.421
7.227
7.224
7.222
4.706
4.549
4.540
4.534
4.527
4.518
3.543
3.531
2.698
1.077
1.066
1.054
10 9 8 7 6 5 4 3 2 1 0 ppm
1.00
1.02
0.99
2.07
0.02
0.03
0.03

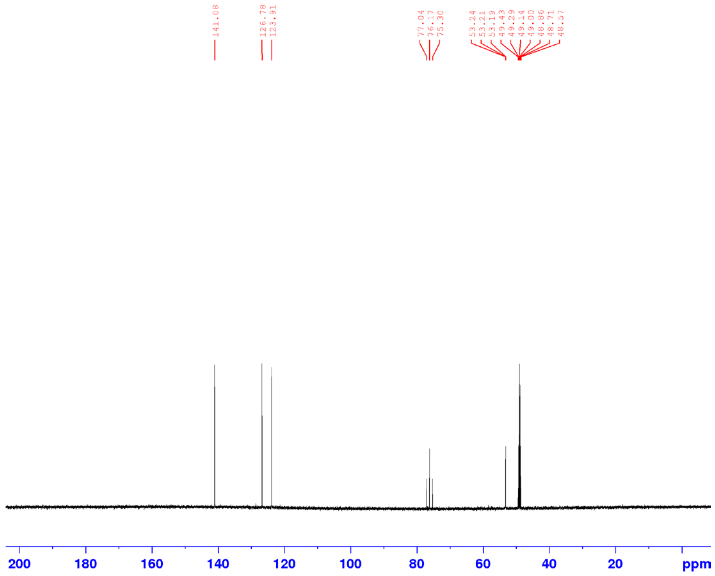
141.08
126.78
123.91
77.04
76.17
75.30
53.24
53.21
53.19
49.43
49.29
49.14
49.00
48.86
48.71
48.57
200 180 160 140 120 100 80 60 40 20 ppm

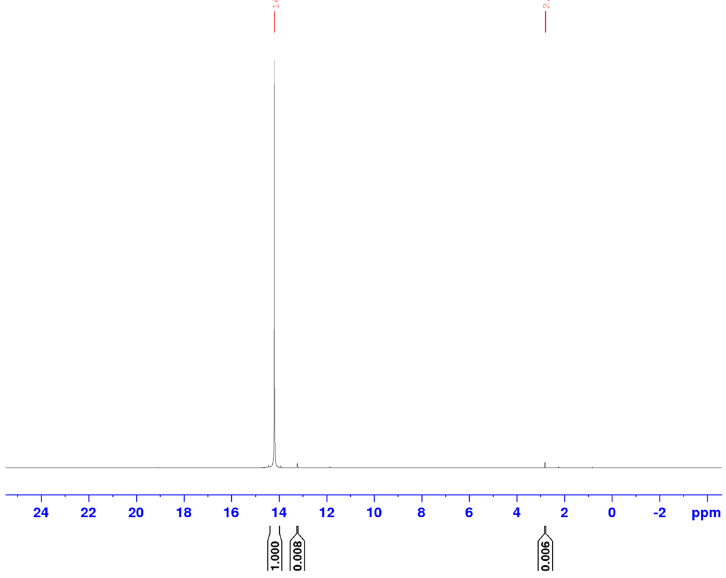
14.19
2.81
24 22 20 18 16 14 12 10 8 6 4 2 0 -2 ppm
1.000
0.008
0.006

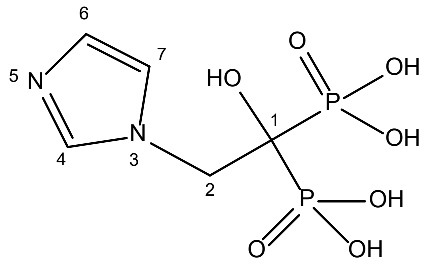

^{1}H NMR (D_2O): δ 7.70 (s, 1H, H-4), 7.21 (s, 1H, H-7), 6.84 (s, 1H, H-6), 4.44 (t, 2H, H-2, $^{3}J_{HP}$ = 9.7). ^{13}C NMR (D_2O, CD_3OD as ref.) δ 141.1 (C-4), 126.8 (C-7), 123.9 (C-6), 76.2 (t, $^{1}J_{CP}$ = 131.5, P-*C*-P, C-1), 53.2 (t, C-2, $^{2}J_{CP}$ = 3.4). ^{31}P NMR (D_2O) δ 16.4. NMR data were consistent to those reported in the literature [29].

Figure S6. ^{1}H (upper), ^{13}C (middle) and ^{31}P (lower) NMR spectra of zoledronic acid monohydrate. Peak assignments are shown as well based on the numbered structural scheme.

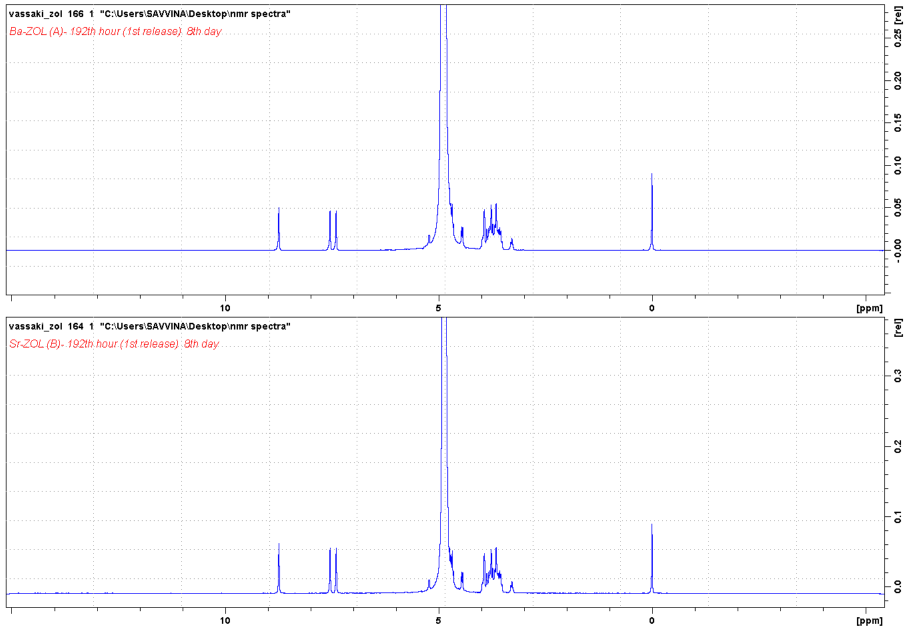

Figure S7. ^{1}H NMR spectra of released zoledronic acid from the Ba-ZOL (above) and Sr-ZOL (below) systems.

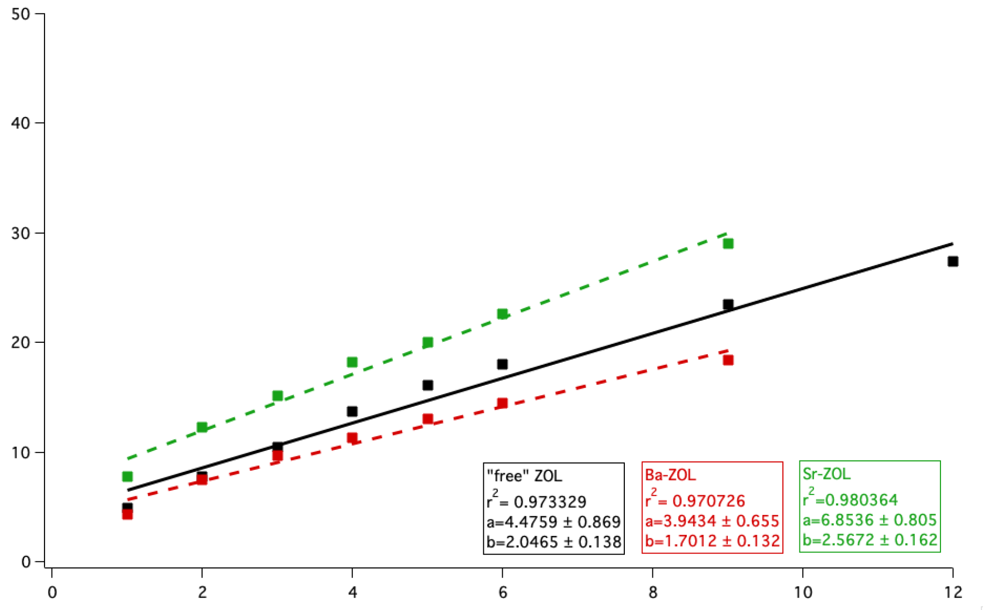

Figure S8. Initial rates of drug release from “free” ZOL, Sr-ZOL and Ba-ZOL.
